# Knowledge, Attitude, and Practices Survey in Greece before the Implementation of Sterile Insect Technique against *Aedes albopictus*

**DOI:** 10.3390/insects12030212

**Published:** 2021-03-02

**Authors:** Angeliki Stefopoulou, Shannon L. LaDeau, Nefeli Syrigou, George Balatsos, Vasileios Karras, Ioanna Lytra, Evangelia Boukouvala, Dimitrios P. Papachristos, Panagiotis G. Milonas, Apostolos Kapranas, Petros Vahamidis, Antonios Michaelakis

**Affiliations:** 1 Benaki Phytopathological Institute, Scientific Directorate of Entomology and Agricultural Zoology, 14561 Kifissia, Greece; a.stefopoulou@bpi.gr (A.S.); g.balatsos@bpi.gr (G.B.); v.karras@bpi.gr (V.K.); i.lytra@bpi.gr (I.L.); d.papachristos@bpi.gr (D.P.P.); p.milonas@bpi.gr (P.G.M.); a.kapranas@bpi.gr (A.K.); or pvachamidis@minagric.gr (P.V.); 2 Cary Institute of Ecosystem Studies, Millbrook, NY 12545, USA; ladeaus@caryinstitute.org; 3 Municipality of Markopoulo Mesogaias, 19003 Markopoulo, Greece; gt@markopoulo.gr (N.S.); evaggeliaboukou@yahoo.com (E.B.); 4 Laboratory of Agronomy, Department of Crop Science, Agricultural University of Athens, 75 Iera Odos, 11855 Athens, Greece

**Keywords:** Asian tiger mosquito, KAP questionnaires, community engagement

## Abstract

**Simple Summary:**

*Aedes albopictus*, also known as the Asian tiger mosquito, tends to breed in various artificial containers frequently found in urban areas. Since urban areas cannot be easily accessed for the application of control measures, community engagement is considered beneficial in vector control. The area of Vravrona, Greece was selected for the implementation of the sterile insect technique (SIT) against *Aedes albopictus* for the first time in Greece. In the current study, a door-to-door campaign was used as a prerelease intervention to raise community awareness about SIT and encourage removal of mosquito habitats in their yards. A KAP (knowledge, attitude, practices) survey was used to collect these data of local community members. Our results demonstrate that using the door-to-door campaign as a prerelease method can raise community awareness, reduce the initial mosquito population, and potentially improve SIT efficacy. The participation of key persons, such as scientific experts and municipality members, in the implementation of the interventions is crucial for the successful engagement of community and may prove important in granting permission to enter their private properties for entomological surveillance.

**Abstract:**

Community involvement in *Aedes albopictus* management can be very efficient and result in raising awareness among citizens. Toward this end, a door-to-door campaign can encourage active community participation in vector control. The current study describes the results of an intervention where a KAP (knowledge, attitude, practices) survey tool was paired with a door-to-door campaign and was implemented as an intervention method in Vravrona area (Attica, Greece) before the release of sterile males (sterile insect technique, SIT) against *Aedes albopictus*. The KAP tool was used to shed light on the knowledge, practices, and attitudes of local community members in order to better prepare and motivate participation in household mosquito control and to assess current understanding of SIT. Each household also received specific information about mosquito source habitat in their own yards at the time of the initial KAP survey. These household data were complemented by standardized mosquito trapping in the municipality. Our findings indicate that citizens’ attitude toward SIT ranged from indecisive to fully supportive, while 77.5% of the respondents agreed that the SIT has many advantages over chemical control methods. Furthermore, the results demonstrate that using the door-to-door campaign as an intervention and prerelease method before SIT can suppress the initial mosquito population and potentially improve its efficacy. Lastly, we show that the presence of local municipality officials during door-to-door visits was associated with increased willingness from the residents to participate in the intervention.

## 1. Introduction

*Aedes albopictus* (Skuse) (Diptera: Culicidae) was first detected in Europe in 1979 (Albania) [1] and was detected in Greece (Corfu and Thesprotia) in 2003 [2]. Since then, *Ae. albopictus* has spread over most of the continental country [3]. Due to its vector competence for various viruses (dengue, chikungunya, yellow fever, etc.), *Ae. albopictus* is globally considered an increasing threat for public health [4].

*Aedes albopictus*, also known as the Asian tiger mosquito, tends to breed in various artificial containers frequently found in urban areas. As private urban areas cannot be accessed by the regional authorities for the application of control measures, there is a consensus that community involvement in vector control could be effective [5]. Education campaigns and community participation are beneficial in vector control [6], as also recommended by the World Health Organization [7]. However, getting people to contribute requires a major effort [8]. Furthermore, it remains uncertain whether education campaigns alone can motivate individuals to participate in mosquito population reduction [9,10,11,12]. KAP questionnaires have been used to test the relationship among the knowledge, attitudes, and practices of residents about local mosquito infestations and demonstrate that, while mosquito-related education can help community-based container management, it is dependent on prior levels of knowledge and concern levels [13]. Other studies suggest that the effect of a multifaceted campaign may result in improved awareness and prevention practices among individuals. Although the presence of scientists inspecting potential habitats alone may be enough to motivate source reduction practices [12,14], a study that used a KAP survey tool regarding dengue prevention concluded that people need not only knowledge but also strong motivation to participate in vector control activities, demonstrating that local knowledge, attitudes, and practices are essential for designing suitable strategies to fit each local context [15,16]. In South Africa, in order to provide baseline information to the communities, a KAP survey on malaria was conducted in 2015 as a first step to present information on the sterile insect technique (SIT) to the community before the application of SIT [17]. The results revealed that a substantial proportion of the community required more information on SIT before its application. Before the 2017 chikungunya outbreak in the Lazio region (Italy), the findings of a KAP questionnaire suggested that citizens were not prepared to face any potential outbreak [18]. According to the findings of the survey, *Ae. albopictus* was still perceived as a nuisance pest rather than a potential vector of diseases. KAP questionnaires were adopted in previous studies to test the relationship among the knowledge, attitudes, and practices of residents [13]. Effective management of *Ae. albopictus* likely depends on the knowledge, attitudes, and practices (KAP) of residents [12].

People can be motivated to reduce mosquito breeding sites in their own backyards for many reasons, including personal experiences and perceptions, and in response to specific information. Disgust has been long recognized as one of the basic human emotions [19,20,21,22] that motivates people to reject or avoid harmful substances and potentially harmful pathogens [17]. Perceptions of disgust towards mosquitoes may be an informative tool of motivation for control activities.

In the current study, we implemented a KAP survey tool paired with a door-to-door campaign as an intervention method in Vravrona area (Attica, Greece) before the release of sterile males (SIT). A door-to-door campaign refers to an intervention to identify potential breeding sites in households. The goal of our campaign was to establish in-person contact with local community members prior to SIT implementation and to collect information regarding community knowledge, attitudes, and perceptions about the risk of mosquito exposure and the SIT.

The SIT is an environment-friendly insect pest management method through which (male) insects are made infertile through irradiation and mass released into a target area. When these sterile males mate with wild females, there is no offspring [23]. The systematic and repeated release of sterile males reduces the target wild insect population over time. The SIT is a method of pest insect control with a strong record of success against a range of agricultural insect pests [24]. Larval control and reduction in breeding sites, both before and during the release of sterile males, is recommended to make SIT sustainable in terms of cost-efficacy [25]. In parallel, a surveillance with ovitraps was implemented in the area to estimate relative abundance of egg-laying mosquitoes.

**Figure 1:**
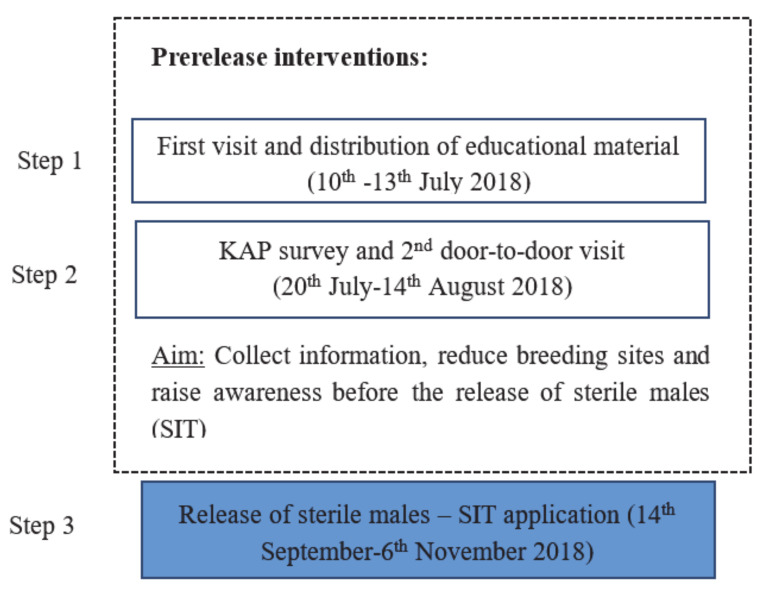
Conceptual diagram. Our current study focuses on the prerelease interventions (Step 1 and Step 2), before application of the sterile insect technique (SIT) [25].

Vravrona area was selected on the basis of (a) its accessible size and its ecological isolation and (b) its proximity to the Athens International Airport, where sterile males were delivered. Figure 1 summarizes the actions and timing of implementation in the control and treated plots before the SIT application, which took place between 14 August and 13 September 2018.

We employed a KAP survey to gain insights into how residents’ knowledge and practices related to mosquitoes varied with demographic variables and to investigate how these factors influence practices aimed at source reduction, including the SIT. The door-to-door campaign was conducted in a twofold approach for the successful implementation of the SIT: (a) to inform inhabitants of our future SIT pilot study and (b) to trigger participation of the public in eliminating breeding sites to reduce mosquito density. To achieve better community engagement, municipality officials were present during the survey and door-to-door campaign. In this context, the KAP survey and door-to-door campaign were used as a prerelease intervention to increase the effectiveness of the SIT application.

## 2. Materials and Methods

### 2.1. Description of Study Site

**Figure 2:**
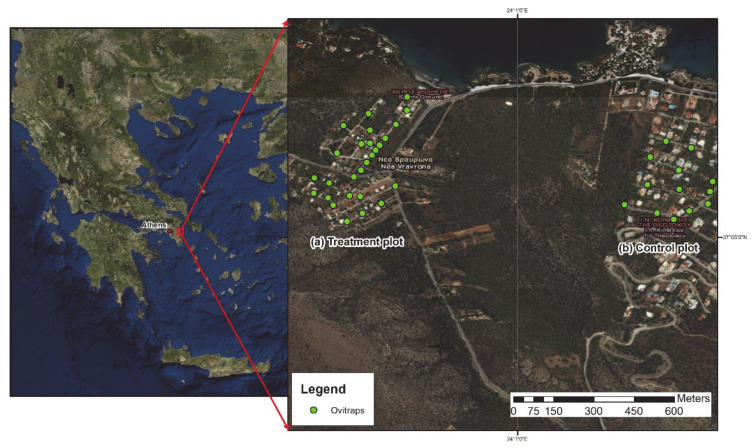
The study area in Vravrona, in Attica region.

The present study employed a KAP survey, a door-to-door campaign to show residents mosquito breeding sites in their own yards, and entomological surveillance to evaluate any changes in the mosquito population during our study. The study was located in the area of Vravrona, in the Municipality of Markopoulo, in Attica Region (Figure 2).

The Vravrona village was used as the treatment plot. It is located in the northeastern part of the Attica region 15 km east of the Athens International Airport, with important tourist and archaeological attractions (centered at 37°55′06.45″ north (N), 24°00′42.78″ east (E)). Its size is 10 ha, isolated from other urban areas by Mediterranean vegetation, while the sea borders the village in the north, and the nearest village, Chamolia, is less than 800 m away to the east. The nearest urban area is 1.6 km south. An untreated control plot (5 ha) was located in the Chamolia village (centered at 37°55′10.58″ N, 24°01′26.78″ Ε). Entomological surveys were conducted in both plots, but the KAP survey and door-to-door identification of breeding sites were only done in the Vravrona village. Each plot had a similar pattern with small family houses and private gardens with Mediterranean vegetation with the same climatic conditions (Figure S1 and Table S1, Supplementary Materials).

Preliminary entomological surveys in both plots confirmed the presence of a population of *Ae. albopictus*. According to the annual epidemiological reports of the National Public Health Organization in Greece, the Municipality of Markopoulo like any other municipality in Greece, has had no previous cases of locally transmitted diseases by *Aedes* species [26,27]. However, there were numerous cases of West Nile virus recorded in many municipalities in Greece (including Municipality of Markopoulo). Only sporadic imported cases of Zika and chikungunya have been reported in Greece [28], while the latest chikungunya outbreaks in Europe were reported in Italy, Spain, and France [29,30].

### 2.2. Distribution of Educational Material and Door-to-Door Intervention

The period of peak abundance of *Ae. albopictus* in this region is September–October [31]. Therefore, all prerelease activities were implemented from 13 July to 14 August. A first visit was made between 10 and 13 July 2018 (Table 1) by two teams of two persons each. Each team consisted of a municipality member and a mosquito expert. The municipality member was from the Environmental Department of the Municipality with knowledge and/or credentials with regard to pest control in the municipality. During this first visit, the teams visited all households (*n* = 91) in the treatment (door-to-door) area, and they talked with the residents, explained the SIT and the aim of the door-to-door intervention, and hand-delivered a leaflet with general information about mosquito bioecology and how to identify and eliminate breeding sites (educational material). The visit took about 10 to 20 min depending on the household. When a resident was absent, the visit was rescheduled for the next day. The KAP survey and the door-to-door intervention were implemented during a second visit, between 20 July and 14 August 2018. The main aim of this intervention was to survey with residents their properties (mainly within their yards) to identify active and potential mosquito breeding sites. The door-to-door intervention was implemented in all households while nearly 44% of the total number of households in the treated plot were chosen at random to fill in the KAP questionnaires (one adult at each household completed the questionnaire). Thus, during the second visit, outside of the KAP questionnaires (see Supplementary Materials) which were filled in at the beginning of the visit, the teams worked in collaboration with the residents to identify all potential habitats and discuss required practices with respect to elimination of breeding sites (door-to-door intervention). The residents were given 15 min to fill in the KAP questionnaires at the beginning of the second visit and, thereafter, another 20 min were spent on average to eliminate all potential breeding sites (e.g., discard water from containers). The numbers of the breeding sites were not recorded and no households were visited in the control plot.

### 2.3. KAP Questionnaires

The questionnaire was based on a previous KAP questionnaire [12] with additional questions to collect information about the respondents’ opinion on SIT and their degree of discomfort toward mosquitoes (disgusting index). Furthermore, the questionnaire collected demographic information such as age, education, gender, ownership status, and presence of children.

*Knowledge level:* To assess the knowledge level of the respondents, the knowledge score of the respondents was estimated. It was based on six questions about mosquito ecology such as where mosquitoes lay their eggs, whether male or female mosquitoes bite humans to get blood, and which diseases are transmitted by mosquitoes. The respondents were also asked to identify the Asian tiger mosquito in a picture with three insects, to choose whether it is an invasive species, and to define which is the most threatening. All correct answers scored 1. Wrong answers scored 0.

*Attitude level:* In this group of questions, respondents were given six statements and were asked to define if they agree or not on a scale from one to five. One responded complete disagreement whereas five responded complete agreement. The respondents were asked (i) to define their level of agreement on whether mosquitoes are beneficial to the ecosystem, (ii) if mosquitoes should be eliminated, (iii) whether it is more significant to get rid of mosquitoes than any other insect, (iv) if they feel threatened by mosquitoes, (v) if they believe that repellants are harmful for human health, and (vi) if the presence of mosquitoes forced them to change their everyday habits (e.g., outdoor activities).

*Protection measures:* This group of questions concerned the types of measures adopted by the respondents to protect themselves from mosquitoes. They were asked to state how often they use chemicals (repellent sprays, tablets, etc.) and nonchemical measures (sieves in windows, bed nets, elimination of breeding sites, removal of stagnant water, etc.), and they were also asked to rate their perceived efficacy.

*Sterile insect technique:* The questionnaire included a section to assess the perception of the respondents toward the SIT. As SIT was applied for the first time in Greece, it was covered extensively by the media and citizens were generally informed about the SIT. Moreover, during our first visit, the respondents had the opportunity to get detailed information about this technique. On the basis of the recommended 10 points for effective community engagement, we provided to the residents’ early information about SIT (with door-to-door campaign) and we worked on building a trust relationship with the community [32]. The suggested 10 points included, among other, rigorous site-selection procedures, early initiation of community engagement activities, establishment of relationships and commitments to build trust with relevant authorities in the community, understanding of community perceptions and attitudes about the proposed research, and secure permission/authorization from the community. At the beginning of the second visit, they were asked their opinion on various aspects of the SIT. Among other questions, they were asked whether they believed that SIT is an effective, realistic method that is safe for humans and the ecosystem and whether they believed that SIT had advantages compared to chemical mosquito control methods.

*Disgusting index:* Disgust was included in the KAP questionnaire since it is argued that it is not an abstract function for general self-protection but motivates behavior and provides solutions to qualitatively distinctive adaptive problems [33]. For this study, the model of Tybur et al. [33] was used according to three functional areas of study of disgust: pathogenic, sexual, and moral. In this questionnaire, we included only items related to pathogen avoidance, which is the “behavioral immune system” that prevents contact with pathogens. We also included questions designed to individuals’ relative mosquito disgust. The questionnaire asked the respondents to rate their disgust toward mosquitoes and other potentially “disgusting” situations. Among other questions, they were asked to state to what degree they feel it is disgusting to kill a mosquito with bare hands or to see blood marks on the wall after killing a mosquito. They were also asked to rate their disgust with respect to thoughts such as the fact that a mosquito has bitten another person or even a mouse before.

### 2.4. Entomological Surveillance with Ovitraps

Oviposition traps, deployed at a density of three ovitraps per ha, were used to monitor *Ae. albopictus* egg density in both plots and to assess the effectiveness of the door-to-door intervention (Figure 2). This resulted in 30 and 15 ovitraps deployed in the treated and control plots, respectively. Ovitraps were deployed close to private households but always in public areas for easy access. In each plot, ovitraps were inspected once a week from May to November 2018. The location of each ovitrap was selected to ensure spatial homogeneity and standardization of environmental conditions that influence the efficacy of the trap [34,35,36]. A geographical information system (ArcGIS, ESRI) was used to divide the treated plot into a grid of 30 rectangular cells and the control plot into 15 rectangular cells. In each rectangular cell, an ovitrap was deployed. The exact location of the ovitrap was recorded with a GPS device and the ovitrap was not moved during the monitoring period. The ovitrap protocol was based on Annex 1 as described in Bellini et al. [36]. The wooden strips (oviposition substrate) were removed from the ovitraps and inspected with a stereoscope in the laboratory, where the total number of eggs was counted. All eggs were hatched and reared until emergence for identification.

### 2.5. Data Analysis

A canonical discriminant function analysis (CDFA) was used to test whether the level of respondents’ knowledge about mosquitoes could be distinguished using a set of predictor variables. The variables assessed through the questionnaires were the following: perceived level of exposure to mosquitoes in their area (C1), level of annoyance (C3), valuing mosquitoes as part of the ecosystem (C10), need to eliminate mosquitoes (C11), significance of mosquitoes over other insects (C12), harmfulness of insect repellences for the human health (C13), altering their plans due to the existence of mosquitoes (C14), feeling threatened by mosquitoes (C15), frequency of use of chemical/nonchemical protection measures and their perceived sufficiency (C16–C19), type of residence (permanent/only for holidays) (C43), education level (C46), and the presence of children living in the same house (C47). For the analysis, the estimated total knowledge score of the respondents was split into four classes. The classes were almost equally divided to count 9–11 records each. The first class included the respondents with high knowledge scores, the second class included those with knowledge scores slightly above average, the third class included scores slightly below average, and the fourth class included scores that correspond to very low knowledge about mosquitoes. The importance of each predictor variable was first assessed by employing a forward stepwise procedure with an F probability threshold of variable introduction set to 0.5. The standardized discriminant function coefficient from each remaining predictor variable was then used to identify the most important ones. The CDFA was performed using the statistical software package SPSS version 16.0 (SPSS Inc. Chicago, IL). Canonical discriminant analysis was then used to identify (1) if the selected knowledge classes can be distinguished by the studied variables, and (2) if yes, which of them were the most important for this discrimination.

To evaluate the relationship between mosquito population abundance and chronological time after initiating control measures, a linear regression analysis was used. We used as, an independent variable, the chronological time (days after the baseline date, which was set as 20 July; see Table 1) and, as a dependent variable, the mean number of eggs collected in ovitraps. The same analysis was done for the control plot.

Quantitative summary statistics and additional comparisons to assess how attitudes and practices were related to each other and to demographic attributes were computed using the R statistical software (version 3.3.1). Welch’s two-sample *t*-test was used to compare the level of respondents’ disgust about mosquitoes relative to other gross things. Pearson correlation coefficients were also calculated to explore the relationship between support for SIT and level of annoyance.

## 3. Results

### 3.1. Characteristics of the Participants

The average age of the participants (*n* = 40) was 53.2 years (degrees of freedom (df) = 39, range = 21–90); 20 (50.0%) were female and 19 (47.5%) were male (one participant did not answer this question). The average age of the women was 53.5 years (range = 25–76) and the average age of the men was 53.3 years (range = 21–90). Fifteen participants had finished secondary education (n = 15, 37.5%), 23 had completed advanced or higher education (57.5%), and two participants did not give details about their educational status. Twenty-six of the participants (65%) were permanent residents in their house, while 14 (35%) visited their residencies for weekends and summer holidays (Table 2). The percentage of participants that had children staying in the same residence was 52.5% (*n* = 21).

**Figure 3:**
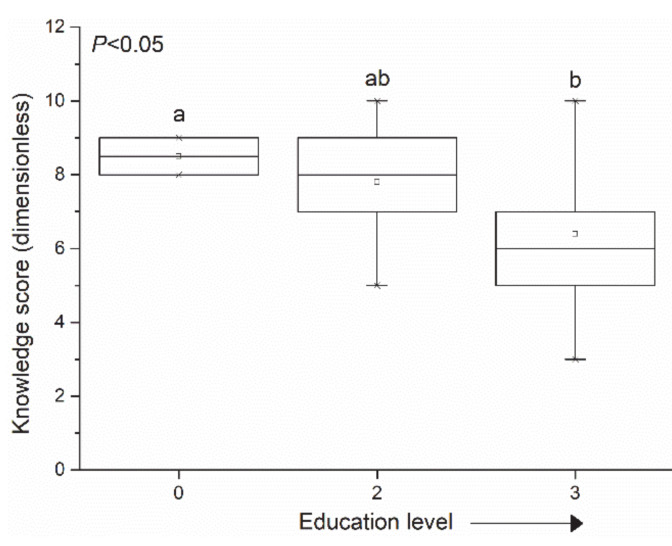
Knowledge score by education level (level 3 stands for higher education, level 2 stands for secondary education, and 0 stands for no education). Broad lines are medians, square open dots are means, boxes show the interquartile range, and whiskers extend to the last data point within 1.5 times the interquartile range. The *p*-value of ANOVA is given. Groups not sharing the same letter are significantly different according to the least significant difference (LSD) test (*p* < 0.05).

People with higher education scored lower in terms of knowledge about mosquitoes (Figure 3) and people that had no formal education scored the highest on mosquito ecology questions.

Most respondents were supportive of the SIT, with a mean response of 4.6 on a scale of 1 (unsupportive) to 5 (fully supportive). None of the demographic characteristics reported were predictive of SIT support (see also Table S2, Supplementary Materials).

### 3.2. KAP: How Knowledge about Mosquitoes Affects the Practices Adopted

To assess their knowledge about mosquitoes, participants answered a number of questions. The maximum knowledge score participants could get was 13 (results: mean = 6.95, range = 3–10).

Almost everyone (92.5%) knew that *Ae. albopictus* is an invasive species and 63% knew that females bite. However, many people were not correct on breeding sites (people chose as many wrong as right answers), and only one person correctly identified the mosquito-borne diseases.

Lower knowledge scores were associated with valuing mosquitoes as part of the ecosystem. People who scored higher on knowledge questions were slightly less likely to agree that mosquitoes had important ecosystem value (*r* = −0.49, *p* = 0.0017). However, there was no clear correlation between this and the respondents’ understanding of vector borne disease or risk.

**Figure 4:**
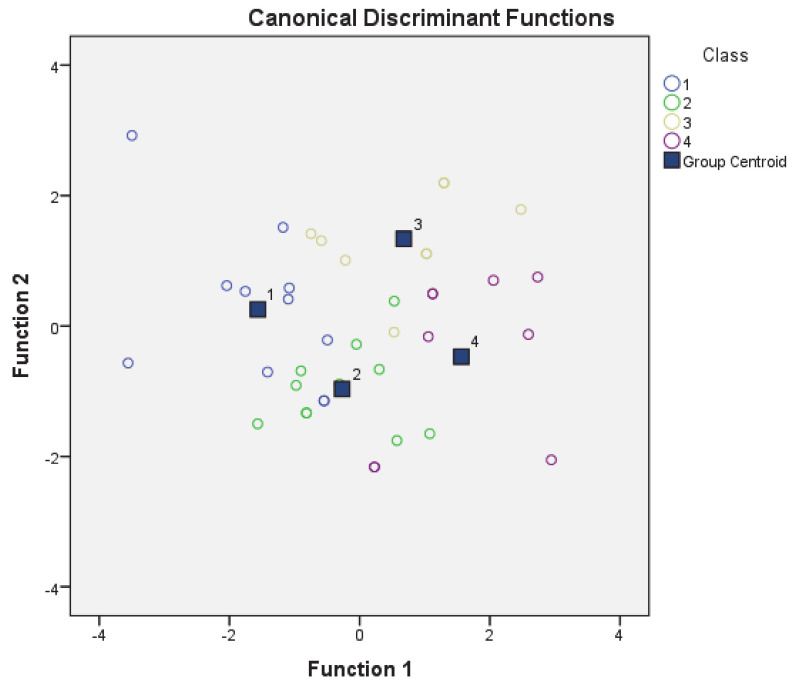
Canonical discriminant analysis (CDA) of the four knowledge groups: (1) group with high knowledge score, (2) group with slightly higher than average knowledge score, (3) group with slightly lower than average knowledge score, and (4) group with low knowledge score.

The use of the forward stepwise procedure retained six predictor variables in the analysis (i.e., C10; C19; C17; C15; C46; C18) and eight were omitted (i.e., C1; C3; C11; C12; C14; C16; C43; C47). The two canonical discriminant functions were statistically significant according to the chi-square statistics (Function 1: *χ*^2^ (18) = 64.579, *p* = 0.000; Function 2: *χ*^2^ (10) = 33.494, *p* = 0.000) (Table 3). Wilk’s’ lambda value of the first function was 0.150, indicating that the proportion of total variability not explained by the knowledge class membership was only 15%. The first two canonical discriminant functions were employed to establish the projective scatter of discriminate scores (Figure 4). It was evident that the centroids for each knowledge class were fairly distinct, suggesting that the selected knowledge classes can be sufficiently distinguished by the remaining variables. Function 1 explained 53.7% of the achieved knowledge scores variance, whereas only 29% of this variance was explained by function 2, which led to a cumulative contribution of 82.7% (Table 4). The first discriminant function was mainly responsible for the distinction of knowledge classes.

According to the magnitude of standardized discriminant coefficients, it was revealed that C15 (feeling threatened by mosquitoes) and C46 (education level) had the highest contribution in function 1, whereas C15 and C18 (frequency of use of chemical protection measures) had the highest contribution in function 2 (Table 3). Therefore, it was clear that the respondents’ knowledge of mosquito ecology was influenced by their feeling of being threatened by mosquitoes, and people who felt threatened tried to improve their knowledge on mosquitoes.

### 3.3. KAP: Practices Adopted by the Participants

The majority of the participants (90%) stated that they take protective measures against mosquitoes, using often or very often (mean 4.25, range = 2–5) biological/nonchemical measures (sieves in windows, bed nets, elimination of breeding sites, removal of stagnant water, etc.) and using chemical measures (repellent sprays, tablets, etc.) at the same frequency (mean = 4.33, range = 1–5). The reported frequency of use was not associated with reported effectiveness of the adopted protective measures (effectiveness vs. nonchemical measures: *r* = −0.15, *p* = 0.36; effectiveness vs. chemical measures: *r* = −0.03, *p* = 0.87). It needs to be emphasized that respondents were asked to define the used frequency on a scale from 1 to 5 with 1 corresponding to “never” and 5 corresponding to “very often”.

People did not agree or disagree with the statement that insect repellent is harmful (mean response = 3.52, range = 1–5), while half of the participants thought that protective measures used were both sufficient and effective.

### 3.4. KAP: What Is People’s Attitude toward SIT

Participants were asked to score SIT between a score of 1 (SIT is a bad idea) and 5 (SIT is a good idea). Among them 37 (92.5%) gave scores above 3 (Table 5). While the majority of participants agreed that SIT is a good idea (mean response = 4.6, range = 2–5), they were less certain that it could be effective (mean response was 3.95, with 1 being “not effective” to 5 being “effective”), and 28 participants (70%) answered with a score above 3. Participants also generally agreed that SIT could be better than chemical control methods (mean response = 4.3, range = 2–5). Among participants, 77.5% agreed that SIT has many advantages over chemical control methods (scored 4 or 5 in a range of 1–5).

Out of a total potential SIT support score of 50, the mean was 42.2 (range = 26–50), indicating that people ranged from indecisive (rates equal to 3) to fully supportive in their responses. Level of annoyance was not associated with support for SIT (*t* = 1.2711, df = 38, *p* = 0.2114). 

### 3.5. Do People Find Mosquitoes Disgusting?

Participants were asked to rate their disgust toward mosquitoes and other gross things from 1 (not at all disgusting) to 5 (overly disgusting). According to the results, people tend to find other gross things more disgusting than mosquitoes (*t* = −1.8199, df = 52.987, *p* = 0.07442, Table 6). Although not statistically different, the mean disgust score for mosquitoes was 3.76 while the respective disgust score for other gross things was 4.18. The observed disgust score was not associated with total knowledge or annoyance level (*t* = 1.5227, df = 38, *p* = 0.1361). It is interesting, nevertheless, that about half of the respondents (55%) found mosquitoes very disgusting but that percentage increased to 62.5% when the statement included their actual biting behavior.

### 3.6. Impact of Door-to-Door Intervention

**Figure 5:**
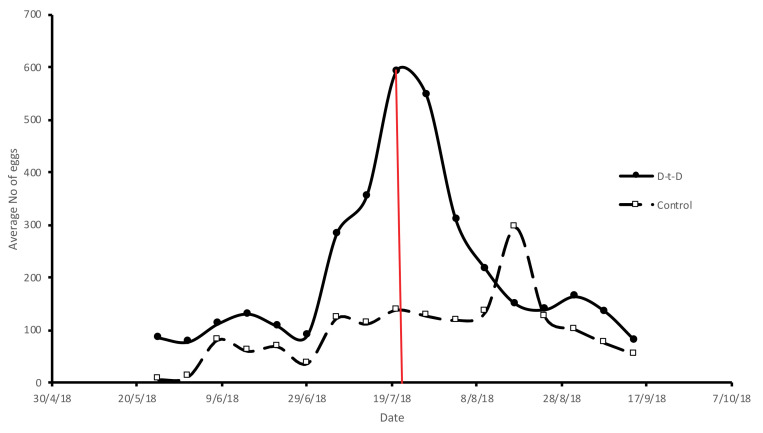
Average of number of eggs in the control and door-to-door (D-t-D) plot for each date. The red vertical line implies the date in which reduction and/or elimination of breeding sites started in D-t-D plot (20 July 2018).

The average number of eggs counted during the first week (before the implementation of the KAP and door-to-door intervention) was 35% greater in the treatment plot than in the control area, indicating a greater initial mosquito population (see Tables S3 and S4, Supplementary Materials, for the raw data). The treatment plot faced a peak in the number of eggs at the beginning of the intervention; however, 2 weeks after the initiation of the door-to-door campaign, the number of eggs significantly decreased and became similar to the total number observed in the control plot (Figure 5). In August 2018, the treatment plot did not show a peak in the eggs, contrary to the control plot. For the entire intervention period, the total number of *Ae. albopictus* eggs in the treatment plot was 40% greater than the total number of eggs in the control plot mainly due to the greater initial population in this plot. The relationship between chronological time and the mean number of eggs was negatively linear in the treatment plot (F_1,7_ = 26.26, *r^2^* = 0.76, *p* = 0.001); however, in the control plot, no significant relationship was found between chronological time and the mean number of eggs (F_1,7_ = 1.05, *r^2^* = 0.13, *p* = 0.341).

## 4. Discussion

The KAP survey provided insight into how the local community of Vravrona perceived the SIT, and it showed the relationship among knowledge, practices, and attitudes of the respondents. The community of Vravrona was generally positive and well informed about the SIT and mosquito control. Because the vast majority was generally supportive, we were probably unable to define any strong demographic predictor of SIT support. Initial house visits in the treatment area may have induced people to reduce breeding sources in their yards, because people became more informed and supportive. This resulted in suppression of the mosquito population before the start of the SIT.

This is the first time in Greece that an SIT field trial against the invasive *Ae. albopictus* has been implemented as an efficient environment-friendly control approach. In addition, this is the first time that a KAP survey and a door-to-door campaign have been implemented in Greece before the release of sterile male *Ae. albopictus*. According to the recommended 10 points for effective community engagement, we provided information about the SIT (through the door-to-door campaign) early to the residents and we developed a relationship of trust with the community [32]. When members of the public are made aware and become well informed about environmental issues, they are likely to become more involved [37,38]. A good communication strategy is essential in SIT field trials for soliciting acceptance of the community [39]. In our study, scientific staff visited all households in the treatment plot and provided information about the SIT, mobilized the residents to engage in source reduction, and talked about their attitudes and perceptions related to the forthcoming SIT field trial. The SIT concept was very well accepted by most of the residents who viewed the SIT as a better idea compared to chemical control methods and 77.5% agreed that SIT has many advantages over chemical control. Furthermore, our findings indicate that their attitude toward SIT ranged from indecisive to fully supportive. We must highlight that, during the sterile male release process, there was no negative reaction of the community. This may be considered as further evidence that the door-to-door awareness campaign was a key-component for the SIT pilot trial. Public acceptance becomes more problematic when other methods such as the release of genetically modified mosquitoes are employed. This was, however, not the case in our study as the mosquitoes were sterilized using X-rays. Every public health program needs community acceptance, especially when direct individual participation is needed [40], as was needed in our project with respect to the elimination of breeding sites.

The KAP survey revealed that, although more than 90% of the respondents knew that *Ae. albopictus* is an invasive species, most of them could not identify the breeding sites and were not aware of which diseases are transmitted by this mosquito species. Therefore, it is probable that the initial visit in the treatment area influenced the behavior of the residents toward breeding site reduction, which resulted in a decline in the egg numbers counted in the area. These findings highlight the need for an intense education campaign among communities to fill knowledge gaps and motivate them toward support for mosquito source reduction. Furthermore, it was clear from the analysis that residents with knowledge scores higher than average felt more threatened by mosquitoes. Lower knowledge scores were associated more with valuing mosquitoes as part of the ecosystem, suggesting a distorted view of a mosquito’s role in the transmission of vector borne diseases. An interesting finding is that people with higher education scored lower in terms of knowledge about mosquito ecology (Figure 3) and people with no formal education scored the highest in this category. In a previous relevant study [11], lower formal education levels were associated with higher motivation among residents. Thus, higher motivation might have covered the gap of lower formal education.

The peak of the number of eggs in the treatment plot at the start of the intervention (elimination/reduction of breeding sites) may be related to the presence of a nearby famous beach that attracts many tourists, especially during summer weekends. Nevertheless, the total number of eggs counted became similar to that of the control plot 2 weeks after the implementation of the door-to-door campaign. Moreover, in mid-August, the number of eggs peaked in the control area but not in the treatment zone. This could be attributed to the earlier reduction and/or elimination of the breeding sites.

Our study also evaluated whether the level of disgust toward mosquitoes was related with factors such as the residents’ practices, the level of discomfort with respect to mosquitoes, and their attitude. Our findings indicate that people find other things more disgusting than mosquitoes and the scores of disgust were not associated with support for SIT or annoyance level. This might be attributed to the fact that Vravrona area has never been exposed to a mosquito-borne disease and, thus, the residents do not feel threatened by *Ae. albopictus*. As we are not aware of any other study in the field of disgust about invasive mosquito species, we cannot state to what degree our results would be consistent with the literature or otherwise.

Another major strength of our study is that door-to-door intervention was implemented in the whole targeted area and not only in a representative number of households. A prerelease suppression of the population is one of the many aspects that makes SIT a powerful tool in modern integrated insect pest-control strategies [41]. Door-to-door intervention was considered as an “obligatory” strategy in the area before the start of the SIT, in order to keep wild male population at low levels and to inform residents of this technique.

Our findings, regarding the average number of eggs, demonstrate that using a door-to-door intervention as a prerelease intervention method can reduce the initial mosquito populations and potentially improve its efficacy. The International Atomic Energy Agency and World Health Organization [42] stress the importance of involving and informing communities ahead of interventions. Our findings corroborate outcomes of previous studies which observed a significant reduction in adult *Ae. albopictus* population after an intense door-to-door education campaign [6,43]. In a previous door-to-door campaign in Greece, it was found that a single visit inspection by a trained mosquito expert can already influence residents’ behavior toward source reduction [12]. A minor limitation was the lack of previous baseline data for mosquito population abundance and dynamics; therefore, we had no information on whether the treatment and control area had similar mosquito population densities. Therefore, we decided to use a linear regression analysis with chronological time as the independent variable. One of the main limitations that we encountered in our trial was the willingness of residents to accept the visits and to answer our questionnaire. To overcome the abovementioned limitation, we decided to include in each team a municipality member together with the mosquito expert. Although all residents accepted the visits, the limitation was mainly related to the acceptance of residents to participate in the survey (questionnaire). The positive influence of the municipality staff member on residents’ support for mosquito management was not measured and can only be reported as an observation. In fact, the participation of a municipality member in the teams acted as mediator between the local community and the scientific team because the community typically knew this person and could trust them. In line with this, Elsigna et al. [44] suggested that a network of key persons can promote the reduction of breeding sites in their neighborhood, while the participation of religious leaders can play a positive role toward enhancing community-based dengue vector management strategies by guiding the local community [44,45,46]. Indeed, our findings converge to the same conclusion as the presence of the municipality members was decisive and resulted in increased willingness from the residents to participate in the intervention.

## 5. Conclusions

The respondents in the questionnaire survey were very positive toward SIT; however, they were less certain that it could be effective. The participation of key persons, such as scientific experts and municipality members, in the implementation of KAP and door-to-door intervention is crucial for the successful engagement of community and maybe more importantly for granting permissions to enter private properties for entomological surveillance. As suggested by our findings, a door-to-door intervention in an isolated site can effectively reduce the initial adult mosquito population, which is considered useful as a prerelease intervention method, tailored to the local characteristics, before implementing the SIT.

## Figures and Tables

**Table 1:** Actions implemented per time period in treatment and control plots. KAP, knowledge, attitude, and practices.

Periods	5 May–6 November 2018	10–13 July 2018	20 July–14 August 2018
Treatment plot (Vravrona)	Entomological surveillance with ovitraps	Communication of the aim of the project to the residents, distribution of the educational material (leaflet)	KAP survey, identification of mosquito breeding sites, and actions for their reduction and/or elimination
Control plot (Chamolia)	Entomological surveillance with ovitraps	None	None

**Table 2:** Questionnaire responses (*n* = 40) on demographic information.

Demographic Information	Number of Residents (%)
Age	
18–45	12 (30.0)
46–60	13 (32.5)
>60	15 (37.5)
Type of settlement	
Permanent residence	26 (65)
Holiday residence	14 (35)
Education level	
Less than high school	0
High school	15 (37.5)
More than high school	23 (57.5)
No answer	2 (5.0)

**Table 3:** Wilk’s’ lambda value per discriminant function. df, degrees of freedom.

Test of Function(s)	Wilks’ Lambda	*χ* ^2^	df	Sig.
1 through 2	0.150	64.579	18	0.000
2	0.373	33.494	10	0.000

**Table 4:** Standardized discriminant function coefficients (functions 1 and 2).

Variables	Function	
	1	2
C10: valuing mosquitoes as part of the ecosystem	0.562	0.490
C15: feeling threaten by mosquitoes	0.762	−0.796
C17: frequency of use of non-chemical protection measures	−0.537	−0.019
C18: frequency of use of chemical protection measures	−0.300	0.683
C19: perceived sufficiency of protective measures	0.433	0.505
C46: education level	0.736	−0.291

**Table 5:** Questionnaire responses on attitude toward SIT.

Response	Number of Residents (%)
It is generally a good idea (*n* = 40)	
0–3 score	3 (7.5)
4–5 score	37 (92.5)
It will be effective (*n* = 40)	
0–3 score	12 (30.0)
4–5 score	28 (70.0)
It has many advantages compared to chemical control methods (*n* = 39)	
0–3 score	8 (20.5)
4–5 score	31 (79.5)
The effectiveness of the method depends on what residents do to manage breeding sources in their home (*n* = 38)	
0–3 score	10 (26.3)
4–5 score	28 (73.7)

**Table 6:** Mosquitoes and disgust (n = 40).

Question	Number of Residents (%)
Do you find mosquitoes disgusting?	
Score 0–3	18 (45)
Score 4–5	22 (55)
Do you find disgusting to kill a mosquito with bare hands?	
Score 0–3	17 (42.5)
Score 4–5	23 (57.5)
Do you find disgusting to see blood marks on the wall, after killing a mosquito?	
Score 0–3	17 (42.5)
Score 4–5	23 (57.5)
Do you find it disgusting to think that, before you, the mosquito has bitten another person?	
Score 0–3	15 (37.5)
Score 4–5	25 (62.5)

## Data Availability

Not applicable.

## Supplementary Materials

The following are available online at https://www.mdpi.com/2075-4450/12/3/212/s1: Figure S1. Average daily temperature (T) and % average daily relative humidity (RH) for the two plots; Table S1. Data for the rainfall and wind speed for the period from 25 June to 13 September (2018) (according to on www.meteo.gr; accessed on 1 March 2021); Table S2. Demographic characteristics and SIT support; Tables S3 and S4. Raw data of ovitraps; KAP Questionnaire (English version).
