# Supplementary material for: Knowledge, Attitude, and Practices Survey in Greece before the Implementation of Sterile Insect Technique against Aedes albopictus
Source: Insects. 2021 Mar 2;12(3):212. doi: 10.3390/insects12030212 (PMC8000271; doi:10.3390/insects12030212)
Supplement: Supplementary file 1 [file insects-12-00212-s001.zip › Figure S1.docx]

Article

Knowledge, Attitude and Practices survey in Greece before the implementation of sterile insect technique against *Aedes albopictus*

Angeliki Stefopoulou ^1^, Shannon L. LaDeau^2^, Nefeli Syrigou^3^, George Balatsos^1^, Vasileios Karras^1^; Ιoanna Lytra ^1^; Evangelia Boukouvala^3^, Dimitrios P. Papachristos^1^, Panagiotis G. Milonas^1^, Apostolos Kapranas^1^, Petros Vahamidis^1,4^ and Antonios Michaelakis^1,*^

^1^ Benaki Phytopathological Institute, Scientific Directorate of Entomology and Agricultural Zoology, 14561, Kifissia; [a.stefopoulou@bpi.gr](mailto:a.stefopoulou@bpi.gr) (A.S); [d.papachristos@bpi.gr](mailto:d.papachristos@bpi.gr) (D.P); [g.balatsos@bpi.gr](mailto:g.balatsos@bpi.gr) (G.B); [v.karras@bpi.gr](mailto:v.karras@bpi.gr) (V.K); [i.lytra@bpi.gr](mailto:i.lytra@bpi.gr) (I.L); [p.milonas@bpi.gr](mailto:p.milonas@bpi.gr) (P.M); [a.kapranas@bpi.gr](mailto:a.kapranas@bpi.gr) (A.K); [pvachamidis@minagric.gr](mailto:pvachamidis@minagric.gr) (P.V); [a.michaelakis@bpi.gr](mailto:a.michaelakis@bpi.gr) (A.M)

^2^ Cary Institute of Ecosystem Studies, Millbrook, New York, United States of America; [ladeaus@caryinstitute.org](mailto:ladeaus@caryinstitute.org)

^3^ Municipality of Markopoulo Mesogaias, Markopoulo, 19003; [gt@markopoulo.gr](mailto:gt@markopoulo.gr) (N.S); [evaggeliaboukou@yahoo.com](mailto:evaggeliaboukou@yahoo.com) (Ε.Β)

^4^ Laboratory of Agronomy, Department of Crop Science, Agricultural University of Athens, 75 Iera Odos, 11855 Athens, Greece; [vahamidis@aua.gr](mailto:vahamidis@aua.gr) (P.V)

***** Correspondence: [a.michaelakis@bpi.gr](mailto:a.michaelakis@bpi.gr); Tel.: +30 210 8180248

**Supplementary Material**

**Figure S1.** Average daily temperature (^o^C) and average daily relative humidity (%) for the control and Door-to-Door (D-t-D) plots.
