# Supplementary material for: Knowledge, Attitude, and Practices Survey in Greece before the Implementation of Sterile Insect Technique against Aedes albopictus
Source: Insects. 2021 Mar 2;12(3):212. doi: 10.3390/insects12030212 (PMC8000271; doi:10.3390/insects12030212)
Supplement: Supplementary file 1 [file insects-12-00212-s001.zip › Table S1.docx]

Article

Knowledge, Attitude and Practices survey in Greece before the implementation of sterile insect technique against *Aedes albopictus*

Angeliki Stefopoulou ^1^, Shannon L. LaDeau^2^, Nefeli Syrigou^3^, George Balatsos^1^, Vasileios Karras^1^; Ιoanna Lytra ^1^; Evangelia Boukouvala^3^, Dimitrios P. Papachristos^1^, Panagiotis G. Milonas^1^, Apostolos Kapranas^1^, Petros Vahamidis^1,4^ and Antonios Michaelakis^1,*^

^1^ Benaki Phytopathological Institute, Scientific Directorate of Entomology and Agricultural Zoology, 14561, Kifissia; [a.stefopoulou@bpi.gr](mailto:a.stefopoulou@bpi.gr) (A.S); [d.papachristos@bpi.gr](mailto:d.papachristos@bpi.gr) (D.P); [g.balatsos@bpi.gr](mailto:g.balatsos@bpi.gr) (G.B); [v.karras@bpi.gr](mailto:v.karras@bpi.gr) (V.K); [i.lytra@bpi.gr](mailto:i.lytra@bpi.gr) (I.L); [p.milonas@bpi.gr](mailto:p.milonas@bpi.gr) (P.M); [a.kapranas@bpi.gr](mailto:a.kapranas@bpi.gr) (A.K); [pvachamidis@minagric.gr](mailto:pvachamidis@minagric.gr) (P.V); [a.michaelakis@bpi.gr](mailto:a.michaelakis@bpi.gr) (A.M)

^2^ Cary Institute of Ecosystem Studies, Millbrook, New York, United States of America; [ladeaus@caryinstitute.org](mailto:ladeaus@caryinstitute.org)

^3^ Municipality of Markopoulo Mesogaias, Markopoulo, 19003; [gt@markopoulo.gr](mailto:gt@markopoulo.gr) (N.S); [evaggeliaboukou@yahoo.com](mailto:evaggeliaboukou@yahoo.com) (Ε.Β)

^4^ Laboratory of Agronomy, Department of Crop Science, Agricultural University of Athens, 75 Iera Odos, 11855 Athens, Greece; [vahamidis@aua.gr](mailto:vahamidis@aua.gr) (P.V)

***** Correspondence: [a.michaelakis@bpi.gr](mailto:a.michaelakis@bpi.gr); Tel.: +30 210 8180248

**Supplementary Material**

**Table S1.** Data for the rainfall and wind speed for the period from 25^th^ of June to 13^th^ of September (2018) (based on [www.meteo.gr](http://www.meteo.gr))

| **Month** | **Day** | **Rain (mm)*** | **Speed (km/h)*** |
| --- | --- | --- | --- |
|  |  |  |  |
| **June** | **25** | 0.0 | 13.2 |
|  | **26** | 3.2 | 7.7 |
|  | **27** | 4.0 | 15.0 |
|  | **28** | 0.0 | 14.3 |
|  | **29** | 0.0 | 18.8 |
|  | **30** | 0.0 | 8.9 |
| **July** | **1** | 0.0 | 5.1 |
|  | **2** | 0.0 | 5.6 |
|  | **3** | 0.0 | 5.0 |
|  | **4** | 0.0 | 5.3 |
|  | **5** | 0.0 | 6.3 |
|  | **6** | 0.0 | 4.7 |
|  | **7** | 0.0 | 5.1 |
|  | **8** | 19.2 | 6.9 |
|  | **9** | 0.0 | 3.5 |
|  | **10** | 0.0 | 3.5 |
|  | **11** | 0.0 | 3.9 |
|  | **12** | 0.0 | 6.4 |
|  | **13** | 0.0 | 7.4 |
|  | **14** | 0.0 | 9.7 |
|  | **15** | 0.0 | 7.2 |
|  | **16** | 0.0 | 4.2 |
|  | **17** | 0.0 | 9.8 |
|  | **18** | 0.0 | 9.5 |
|  | **19** | 0.0 | 6.9 |
|  | **20** | 0.0 | 6.6 |
|  | **21** | 0.0 | 4.0 |
|  | **22** | 0.0 | 7.9 |
|  | **23** | 0.0 | 18.3 |
|  | **24** | 0.4 | 5.0 |
|  | **25** | 1.2 | 4.3 |
|  | **26** | 0.0 | 3.5 |
|  | **27** | 0.0 | 4.3 |
|  | **28** | 17.2 | 3.9 |
|  | **29** | 25.4 | 4.2 |
|  | **30** | 0.0 | 6.8 |
|  | **31** | 0.0 | 6.1 |
| **August** | **1** | 0.0 | 6.1 |
|  | **2** | 0.0 | 8.5 |
|  | **3** | 0.0 | 15.6 |
|  | **4** | 0.0 | 16.4 |
|  | **5** | 0.0 | 14.6 |
|  | **6** | 0.0 | 10.5 |
|  | **7** | 0.0 | 8.9 |
|  | **8** | 0.0 | 11.3 |
|  | **9** | 0.0 | 16.6 |
|  | **10** | 0.0 | 19.3 |
|  | **11** | 0.0 | 19.2 |
|  | **12** | 0.0 | 15.4 |
|  | **13** | 0.0 | 12.7 |
|  | **14** | 0.0 | 9.3 |
|  | **15** | 0.0 | 7.1 |
|  | **16** | 0.0 | 5.3 |
|  | **17** | 0.0 | 4.3 |
|  | **18** | 0.0 | 9.5 |
|  | **19** | 0.0 | 15.0 |
|  | **20** | 0.0 | 17.1 |
|  | **21** | 0.0 | 13.2 |
|  | **22** | 0.0 | 10.3 |
|  | **23** | 0.0 | 10.6 |
|  | **24** | 0.0 | 10.1 |
|  | **25** | 0.0 | 3.5 |
|  | **26** | 0.0 | 4.5 |
|  | **27** | 0.0 | 6.0 |
|  | **28** | 1.2 | 8.9 |
|  | **29** | 0.0 | 14.6 |
|  | **30** | 0.0 | 11.7 |
|  | **31** | 0.0 | 7.7 |
| **September** | **1** | 0.0 | 7.6 |
|  | **2** | 0.0 | 8.0 |
|  | **3** | 0.0 | 6.9 |
|  | **4** | 0.0 | 5.8 |
|  | **5** | 0.0 | 4.2 |
|  | **6** | 0.0 | 3.5 |
|  | **7** | 0.6 | 5.1 |
|  | **8** | 7.2 | 3.2 |
|  | **9** | 0.0 | 5.6 |
|  | **10** | 0.0 | 6.0 |
|  | **11** | 0.0 | 4.5 |
|  | **12** | 0.0 | 9.0 |
|  | **13** | 0.0 | 11.4 |
