# Supplementary material for: Knowledge, Attitude, and Practices Survey in Greece before the Implementation of Sterile Insect Technique against Aedes albopictus
Source: Insects. 2021 Mar 2;12(3):212. doi: 10.3390/insects12030212 (PMC8000271; doi:10.3390/insects12030212)
Supplement: Supplementary file 1 [file insects-12-00212-s001.zip › Tables S3_S4.docx]

Article

Knowledge, Attitude and Practices survey in Greece before the implementation of sterile insect technique against *Aedes albopictus*

Angeliki Stefopoulou ^1^, Shannon L. LaDeau^2^, Nefeli Syrigou^3^, George Balatsos^1^, Vasileios Karras^1^; Ιoanna Lytra ^1^; Evangelia Boukouvala^3^, Dimitrios P. Papachristos^1^, Panagiotis G. Milonas^1^, Apostolos Kapranas^1^, Petros Vahamidis^1,4^ and Antonios Michaelakis^1,*^

^1^ Benaki Phytopathological Institute, Scientific Directorate of Entomology and Agricultural Zoology, 14561, Kifissia; [a.stefopoulou@bpi.gr](mailto:a.stefopoulou@bpi.gr) (A.S); [d.papachristos@bpi.gr](mailto:d.papachristos@bpi.gr) (D.P); [g.balatsos@bpi.gr](mailto:g.balatsos@bpi.gr) (G.B); [v.karras@bpi.gr](mailto:v.karras@bpi.gr) (V.K); [i.lytra@bpi.gr](mailto:i.lytra@bpi.gr) (I.L); [p.milonas@bpi.gr](mailto:p.milonas@bpi.gr) (P.M); [a.kapranas@bpi.gr](mailto:a.kapranas@bpi.gr) (A.K); [pvachamidis@minagric.gr](mailto:pvachamidis@minagric.gr) (P.V); [a.michaelakis@bpi.gr](mailto:a.michaelakis@bpi.gr) (A.M)

^2^ Cary Institute of Ecosystem Studies, Millbrook, New York, United States of America; [ladeaus@caryinstitute.org](mailto:ladeaus@caryinstitute.org)

^3^ Municipality of Markopoulo Mesogaias, Markopoulo, 19003; [gt@markopoulo.gr](mailto:gt@markopoulo.gr) (N.S); [evaggeliaboukou@yahoo.com](mailto:evaggeliaboukou@yahoo.com) (Ε.Β)

^4^ Laboratory of Agronomy, Department of Crop Science, Agricultural University of Athens, 75 Iera Odos, 11855 Athens, Greece; [vahamidis@aua.gr](mailto:vahamidis@aua.gr) (P.V)

***** Correspondence: [a.michaelakis@bpi.gr](mailto:a.michaelakis@bpi.gr); Tel.: +30 210 8180248

**Supplementary Material**

**Table S3.** Raw data of ovitraps in treatment plot. Total No of eggs/ovitrap/week. The missing values are indicated as ND (no data).

| **No of week** | 29 | 30 | 31 | 32 | 33 | 34 | 35 | 36 | 37 |
| --- | --- | --- | --- | --- | --- | --- | --- | --- | --- |
| **Period** | 13/7/2018 - 20/7/2018 | 20/7/2018 - 27/7/2018 | 27/7/2018 - 3/8/2018 | 3/8/2018 - 10/8/2018 | 10/8/2018 - 17/8/2018 | 17/8/2018 - 24/8/2018 | 24/8/2018 - 31/8/2018 | 31/8/2018 - 7/9/2018 | 7/9/2018 - 14/9/2018 |
| **No of ovitrap** |  |  |  |  |  |  |  |  |  |
| Β1 | 64 | 156 | 0 | 0 | 1 | 1 | 6 | 45 | 2 |
| Β2 | 341 | 518 | 41 | 109 | 33 | 3 | 116 | 72 | 20 |
| Β3 | 553 | 620 | ND | 558 | 108 | 90 | 190 | 0 | 40 |
| Β4 | 866 | 899 | 296 | 383 | 239 | 92 | 258 | 240 | 94 |
| Β5 | 1107 | 298 | 204 | 346 | 248 | 202 | 262 | 9 | 103 |
| Β6 | 2254 | 330 | 241 | 59 | 35 | 122 | 120 | 114 | 59 |
| Β7 | 202 | ND | 0 | ND | 96 | 522 | 57 | 145 | 73 |
| Β8 | 162 | 1084 | 622 | 181 | 291 | 138 | 174 | 157 | 132 |
| Β9 | 442 | 887 | 515 | 193 | 147 | 35 | 156 | 209 | 107 |
| Β10 | 408 | 937 | 287 | 68 | 10 | 28 | 120 | 15 | 101 |
| Β11 | 405 | 88 | 419 | 26 | 354 | 12 | 24 | 139 | 94 |
| Β12 | 555 | 497 | 11 | 161 | 24 | 28 | 12 | 35 | 46 |
| Β13 | 0 | 3 | 47 | 0 | 0 | 4 | 37 | 2 | 12 |
| Β14 | 0 | 0 | ND | 5 | 33 | 87 | 44 | 33 | 0 |
| Β15 | 44 | 426 | 297 | 155 | 41 | 271 | 179 | 173 | 68 |
| Β16 | 9 | 71 | 48 | 9 | 42 | 47 | 108 | 71 | 12 |
| Β17 | 46 | 121 | 148 | 66 | 18 | 79 | 38 | 26 | 5 |
| Β18 | 19 | 23 | 56 | 14 | 34 | 17 | 113 | 6 | 0 |
| Β19 | 133 | 166 | 32 | 82 | 40 | 61 | 50 | ND | 8 |
| Β20 | 60 | 193 | 111 | 0 | 154 | 95 | 69 | 15 | 80 |
| Β21 | 388 | 318 | 295 | 339 | 102 | 105 | 175 | 76 | 44 |
| Β22 | 27 | 42 | 94 | 44 | 1 | 1 | 15 | 30 | 0 |
| Β23 | 161 | 43 | 90 | 0 | 103 | 0 | 77 | 77 | 3 |
| Β24 | 31 | 13 | 68 | 24 | 46 | 0 | 6 | 35 | 16 |
| Β25 | 24 | 5 | 46 | 0 | 0 | 19 | 17 | 11 | 16 |
| Β26 | 101 | 11 | 132 | 28 | 7 | 31 | 0 | 151 | 13 |
| Β27 | 68 | 61 | 49 | 77 | 12 | 15 | 67 | 69 | 63 |
| Β28 | 182 | 67 | 220 | 9 | 22 | 30 | 23 | 22 | 6 |
| Β29 | 216 | 46 | 0 | 61 | 26 | 19 | 15 | 23 | 0 |
| Β30 | ND | ND | 112 | 237 | 58 | 3 | 11 | 37 | 44 |

**Table S4.** Raw data of ovitraps in control plot. Total No of eggs/ovitrap/week. The missing values are indicated as ND (no data).

| **No of week** | 29 | 30 | 31 | 32 | 33 | 34 | 35 | 36 | 37 |
| --- | --- | --- | --- | --- | --- | --- | --- | --- | --- |
| **Period** | 13/7/2018 - 20/7/2018 | 20/7/2018 - 27/7/2018 | 27/7/2018 - 3/8/2018 | 3/8/2018 - 10/8/2018 | 10/8/2018 - 17/8/2018 | 17/8/2018 - 24/8/2018 | 24/8/2018 - 31/8/2018 | 31/8/2018 - 7/9/2018 | 7/9/2018 - 14/9/2018 |
| **No of ovitrap** |  |  |  |  |  |  |  |  |  |
| B31 | 269 | ND | 0 | 3 | 153 | 25 | 81 | 0 | 0 |
| B32 | 295 | 174 | 226 | 226 | 633 | 256 | 94 | 109 | 31 |
| B33 | 148 | 209 | 277 | 130 | 357 | 109 | 75 | 15 | 48 |
| B34 | 100 | 120 | 143 | 131 | 351 | 84 | 149 | 213 | 70 |
| B35 | 80 | 107 | 190 | 113 | 251 | 215 | 207 | 312 | 168 |
| B36 | 75 | 46 | 44 | 123 | 274 | 240 | 214 | 79 | 78 |
| B37 | 260 | 138 | 138 | 271 | 582 | 276 | 132 | 58 | 113 |
| B38 | 122 | 216 | 147 | 461 | 457 | 258 | 144 | 72 | 189 |
| B39 | 170 | 255 | 145 | 25 | 152 | 106 | 85 | 99 | 20 |
| B40 | 135 | ND | 21 | 129 | 113 | 116 | 105 | 23 | 17 |
| B41 | 112 | 141 | 81 | 39 | 348 | 92 | 129 | 25 | 7 |
| B42 | 109 | 34 | 56 | 18 | 38 | 45 | 84 | 31 | 45 |
| B43 | 38 | 0 | 2 | ND | ND | 0 | 3 | 0 | 0 |
| B44 | 71 | ND | ND | ND | ND | 2 | 0 | 8 | 18 |
| B45 | 95 | 86 | 195 | 88 | 131 | 36 | 19 | 87 | 13 |
