# Supplementary material for: Knowledge, Attitude, and Practices Survey in Greece before the Implementation of Sterile Insect Technique against Aedes albopictus
Source: Insects. 2021 Mar 2;12(3):212. doi: 10.3390/insects12030212 (PMC8000271; doi:10.3390/insects12030212)
Supplement: Supplementary file 1 [file insects-12-00212-s001.zip › KAP_questionnaire_en.doc]

Article

Knowledge, Attitude and Practices survey in Greece before the implementation of sterile insect technique against *Aedes albopictus*

Angeliki Stefopoulou ^1^, Shannon L. LaDeau^2^, Nefeli Syrigou^3^, George Balatsos^1^, Vasileios Karras^1^; Ιoanna Lytra ^1^; Evangelia Boukouvala^3^, Dimitrios P. Papachristos^1^, Panagiotis G. Milonas^1^, Apostolos Kapranas^1^, Petros Vahamidis^1,4^ and Antonios Michaelakis^1,*^

^1^	Benaki Phytopathological Institute, Scientific Directorate of Entomology and Agricultural Zoology, 14561, Kifissia; [a.stefopoulou@bpi.gr](mailto:a.stefopoulou@bpi.gr) (A.S); [d.papachristos@bpi.gr](mailto:d.papachristos@bpi.gr) (D.P); [g.balatsos@bpi.gr](mailto:g.balatsos@bpi.gr) (G.B); [v.karras@bpi.gr](mailto:v.karras@bpi.gr) (V.K); [i.lytra@bpi.gr](mailto:i.lytra@bpi.gr) (I.L); [p.milonas@bpi.gr](mailto:p.milonas@bpi.gr) (P.M); [a.kapranas@bpi.gr](mailto:a.kapranas@bpi.gr) (A.K); [pvachamidis@minagric.gr](mailto:pvachamidis@minagric.gr) (P.V); [a.michaelakis@bpi.gr](mailto:a.michaelakis@bpi.gr) (A.M)

^2^	Cary Institute of Ecosystem Studies, Millbrook, New York, United States of America; [ladeaus@caryinstitute.org](mailto:ladeaus@caryinstitute.org)

^3^	Municipality of Markopoulo Mesogaias, Markopoulo, 19003; [gt@markopoulo.gr](mailto:gt@markopoulo.gr) (N.S); [evaggeliaboukou@yahoo.com](mailto:evaggeliaboukou@yahoo.com) (Ε.Β)

^4^	Laboratory of Agronomy, Department of Crop Science, Agricultural University of Athens, 75 Iera Odos, 11855 Athens, Greece; [vahamidis@aua.gr](mailto:vahamidis@aua.gr) (P.V)

*****	Correspondence: [a.michaelakis@bpi.gr](mailto:a.michaelakis@bpi.gr); Tel.: +30 210 8180248

**Supplementary Material**

KAP Questionnaire (English version)

**1. Are there any mosquitoes in your area?**

| 1 | 2 | 3 | 4 | 5 |
| --- | --- | --- | --- | --- |
| No | A few | Many | Quite a lot | Excessive |

2. In Case of a positive answer, then they are mostly seen:

| During daytime | (1) |
| --- | --- |
| During night | (2) |
| Both | (3) |

**3. Level of your annoyance:**

| 1 | 2 | 3 | 4 | 5 |
| --- | --- | --- | --- | --- |
| None | A little | Some | Great | Extreme |

**4. Where do the mosquitoes lay eggs? (0-> not selected, 1->selected)**

| 4.1.Standing water |  | 4.4. Mud |  | 4.7. Grass-plants |  |
| --- | --- | --- | --- | --- | --- |
| 4.2. soil |  | 4.5. Flowerpot |  | 4.8. creeks |  |
| 4.3. drainage |  | 4.6. Old (used) tires |  | 4.9. External flowerpots |  |

**5. Do you know which mosquitoes sting?**

| Males | (1) | Females | (2) | Both | **(3)** |
| --- | --- | --- | --- | --- | --- |

**6. The Asian mosquito tiger is considered:**

| Local species | (1) | New, non-local species | (2) |
| --- | --- | --- | --- |

**7. Which of the following is the mosquito ‘tiger’?**

1. □	2. □	3. □

**8. Which of the above is more threatening ?**

| □ | □ | □ | □ |
| --- | --- | --- | --- |
| the first(1) | the second (2) | the third(3) | None(4) |

**9. Which diseases do the mosquitoes carry? (0-> not selected, 1->selected)**

| 9.1. Dengue |  | 9.4. chikungunya |  | 9.7 Bird flu |  |
| --- | --- | --- | --- | --- | --- |
| 9.2. Measles |  | 9.5. Aids |  | 9.8 zika |  |
| 9.3. Malaria |  | 9.6. Tuberculosis |  | 9.9 West Nile virus |  |

**In what level do you** agree or disagree with the following statements?

| 10 Mosquitoes are beneficial part of the ecosystem | Completely disagree | 1 | 2 | 3 | 4 | 5 | Completely agree |
| --- | --- | --- | --- | --- | --- | --- | --- |
| 11. Mosquitoes must be eliminated from the human societies | Completely disagree | 1 | 2 | 3 | 4 | 5 | Completely agree |
| 12. It is more significant to get rid of the mosquitoes than any other insect | Completely disagree | 1 | 2 | 3 | 4 | 5 | Completely agree |
| 13. Insect repellents are harmful for human health | Completely disagree | 1 | 2 | 3 | 4 | 5 | Completely agree |
| 14. The existence of mosquitoes has forced me to alter my plans | Completely disagree | 1 | 2 | 3 | 4 | 5 | Completely agree |
| 15.I feel threatened by the mosquitoes | Completely disagree | 1 | 2 | 3 | 4 | 5 | Completely agree |

**16. Do you or any member of your family take measures of protection against mosquitoes?**

**Yes (1)	No (2)**

**17. When you want to be protected from mosquitoes on your property, how often do you choose biological/non-chemical measures of protection (sieves in windows, bed nets, elimination of breeding sites etc)?**

| 1 | 2 | 3 | 4 | 5 |
| --- | --- | --- | --- | --- |
| **Never** | **Rarely** | **Sometimes** | **Often** | **Very often** |

**18. Respectively, when you want to be protected from mosquitoes on your property, how often do you choose measures of protection that are based on chemicals**?

| 1 | 2 | 3 | 4 | 5 |
| --- | --- | --- | --- | --- |
| **Never** | **Rarely** | **Sometimes** | **Often** | **Very often** |

**19. When you take measures of protection against mosquitoes, do you feel that they are sufficient and effective?**

**Yes (1)	No (2)**

**20. Which methods of mosquitoes’ population control, is more effective?**

| Natural | (1) | Chemicals | (2) | Both | (3) |
| --- | --- | --- | --- | --- | --- |

| **21. Do you find mosquitoes disgusting?** | Not at all disgusting | 1 | 2 | 3 | 4 | 5 | Overly disgusting |
| --- | --- | --- | --- | --- | --- | --- | --- |

**These are a few suggestions that describe a series of situations. Please rate how disgusting do you consider each of the proposals described:**

**22.** In what degree do you find disgusting to kill a mosquito with bare hands?

| Not at all disgusting | 1 | 2 | 3 | 4 | 5 | Overly disgusting |
| --- | --- | --- | --- | --- | --- | --- |

**23. In what degree do you find disgusting to see blood marks on the wall, after killing a mosquito?**

| Not at all disgusting | 1 | 2 | 3 | 4 | 5 | Overly disgusting |
| --- | --- | --- | --- | --- | --- | --- |

**24. In what degree do you find it disgusting to think,** that before you, the mosquito has bitten another person?

| Not at all disgusting | 1 | 2 | 3 | 4 | 5 | Overly disgusting |
| --- | --- | --- | --- | --- | --- | --- |

**25. In what degree do you find it disgusting to think, that before you,** the mosquito has bitten a mouse?

| Not at all disgusting | 1 | 2 | 3 | 4 | 5 | Overly disgusting |
| --- | --- | --- | --- | --- | --- | --- |

**26. In what degree do you find disgusting to step on dog feces**?

| Not at all disgusting | 1 | 2 | 3 | 4 | 5 | Overly disgusting |
| --- | --- | --- | --- | --- | --- | --- |

**27. In what degree do you it find disgusting to sit next to someone who has red sores on his arm.**

| Not at all disgusting | 1 | 2 | 3 | 4 | 5 | Overly disgusting |
| --- | --- | --- | --- | --- | --- | --- |

**28. In what degree do you find it disgusting to shake hands with an unknown person who has** sweaty palms.

| Not at all disgusting | 1 | 2 | 3 | 4 | 5 | Overly disgusting |
| --- | --- | --- | --- | --- | --- | --- |

**29.In what degree do you find it disgusting to see a little mold in food leftovers, in your refrigerator?**

| Not at all disgusting | 1 | 2 | 3 | 4 | 5 | Overly disgusting |
| --- | --- | --- | --- | --- | --- | --- |

**30. In what degree do you find it disgusting to stand next to someone who stinks?**

| Not at all disgusting | 1 | 2 | 3 | 4 | 5 | Overly disgusting |
| --- | --- | --- | --- | --- | --- | --- |

**31. In what degree do you find it disgusting to see a cockroach crossing the floor?**

| Not at all disgusting | 1 | 2 | 3 | 4 | 5 | Overly disgusting |
| --- | --- | --- | --- | --- | --- | --- |

**32. In what degree do you find it disgusting to unintentionally touch the bleeding scar of a person?**

| Not at all disgusting | 1 | 2 | 3 | 4 | 5 | Overly disgusting |
| --- | --- | --- | --- | --- | --- | --- |

**Informative about the application of the Sterile Insect Technique (SIT) to the Asian tiger mosquito**

We inform you that in the Benakio Phytopathological Institute, in collaboration with the Hellenic Atomic Energy Commission and the University of Thessaly (Laboratory of Entomology and Applied Zoology), a project is being carried out, that relates to the application of the Sterile Insect Technique (SIT) against the Asian tiger mosquito (Aedes albopictus).

In the context of the above project and because the Aedes albopictus mosquito species is already established in many regions of Greece, preliminary steps have been taken, to apply - for the first time in our country - the technique of releasing sterile insects Sterile Insect Technique (SIT) against this mosquito species. The evaluation of the "SIT" method through a pilot project in our country, is the primary objective of the project to help in order of a future Aedes albopictus population repression in Greece. In addition, the aim of this pilot application is to combine the SIT strategy with other mosquito management methods.

It is noted, that the sterile mosquitoes to be unleashed are males only (which do not sting human) and have been sterilized by methods based on the use of radioactivity, mainly X-rays generated by machines similar to those in hospitals and medical centers and not with genetic methods.

The area that has been chosen for the first time pilot assessment of this new method in Greece, is Vravrona (Municipality of Markopoulo) and the implementation period is during September - October 2018.

***It would be very useful to tell us your opinion about this new method:***

Do you think that the method of releasing sterilized male insects to fight the Asian tiger mosquito:

| 33. Is generally a bad idea | 1 | 2 | 3 | 4 | 5 | Is generally a good idea |
| --- | --- | --- | --- | --- | --- | --- |
| 34. Will not be effective | 1 | 2 | 3 | 4 | 5 | Will be totally effective |
| 35. It is silly | 1 | 2 | 3 | 4 | 5 | It is smart |
| 36. It would not be accepted by Greek society | 1 | 2 | 3 | 4 | 5 | It would be accepted by Greek society |
| 37. It has many drawbacks compared to chemical control methods | 1 | 2 | 3 | 4 | 5 | It has many advantages over chemical control methods |
| 38.It is harmful to the ecosystem | 1 | 2 | 3 | 4 | 5 | It is safe for the ecosystem |
| 39. It is harmful to humans | 1 | 2 | 3 | 4 | 5 | It is safe for humans |
| 40. It is not necessary | 1 | 2 | 3 | 4 | 5 | It is necessary |
| 41. It is not realistic at all | 1 | 2 | 3 | 4 | 5 | It is absolutely realistic |
| 42.The effectiveness of the method is independent of what residents do to manage mosquito nests in their home | 1 | 2 | 3 | 4 | 5 | The success of the method is influenced by the meticulousness of the methods used by residents to manage their mosquito nests in their home |

***Finally, we would like to know some details about you, for demographic reasons.***

We remind you that all data collected are absolutely confidential.

43. you live in this settlement:

permantly (2)	for holidays (1)

44. You are

Male	(1)	Female (2)	Without answer (3)

45. How old are you?

46. What is your education level?

| Primary education | (1) | Upper or Higher Education | (3) |
| --- | --- | --- | --- |
| Secondary education | (2) | No Answer | (4) |

47. Do you have children or do you have children living in the same house with you?

Yes (1)	No (2)

Date of completion:……………………………… ……

## Thank you for your time.

CODE) : ………
